# Effects of Environmental Exposure on Host and Microbial Metabolism

**DOI:** 10.3390/metabo15100646

**Published:** 2025-09-26

**Authors:** Bei Gao, Pengcheng Tu

**Affiliations:** 1 School of Environmental Science and Engineering, Nanjing University of Information Science and Technology, Nanjing 210044, China; bgao@nuist.edu.cn; 2 Zhejiang Provincial Center for Disease Control and Prevention, 3399 Binsheng Road, Hangzhou 310051, China

Trillions of microorganisms are living in our gastrointestinal tract, known as the gut microbiota, which plays an essential role in human health and disease. The gut microbiota could be affected by many factors, including environmental exposure. Disruptions of the gut microbiota by environmental exposure may exert adverse effects on human health by affecting host metabolism, intrinsic metabolism in the gut microbiota, and/or gut microbiota–host co-metabolism. This Special Issue, “Effects of Environmental Exposure on Host and Microbial Metabolism”, contains six original research articles and five review articles, which covers the effects of both emerging environmental pollutants, including microplastics, per- and polyfluoroalkyl substances (PFASs), bisphenol S (BPS), and traditional fluoride and fungicide epoxiconazole (EPX) contaminants, as well as dietary factors such as non/low-caloric artificial sweeteners (NAS), probiotics, prebiotics, and natural compounds. The contributions are listed below.

1. Tu, P.; Xue, J.; Niu, H.; Tang, Q.; Mo, Z.; Zheng, X.; Wu, L.; Chen, Z.; Cai, Y.; Wang, X. Deciphering Gut Microbiome Responses upon Microplastic Exposure via Integrating Metagenomics and Activity-Based Metabolomics. *Metabolites* **2023**, *13*, 530. https://doi.org/10.3390/metabo13040530.
2. Niu, H.; Yang, Y.; Zhou, Y.; Ma, X.; Ding, Z.; Xu, M.; Wu, L.; Li, X.; Xing, M.; Zhang, Q.; et al. Differential Impacts of Environmentally Relevant Microplastics on Gut Barrier Integrity in Mice Fed High-Fat Diet Versus Normal Chow Diet. *Metabolites* **2025**, *15*, 557. https://doi.org/10.3390/metabo15080557.
3. Xu, M.; Niu, H.; Wu, L.; Xing, M.; Mo, Z.; Chen, Z.; Li, X.; Lou, X. Impact of Microplastic Exposure on Blood Glucose Levels and Gut Microbiota: Differential Effects under Normal or High-Fat Diet Conditions. *Metabolites* **2024**, *14*, 504. https://doi.org/10.3390/metabo14090504.
4. Niu, H.; Liu, S.; Jiang, Y.; Hu, Y.; Li, Y.; He, L.; Xing, M.; Li, X.; Wu, L.; Chen, Z.; et al. Are Microplastics Toxic? A Review from Eco-Toxicity to Effects on the Gut Microbiota. *Metabolites* **2023**, *13*, 739. https://doi.org/10.3390/metabo13060739.
5. Cox, A.; Nowshad, F.; Callaway, E.; Jayaraman, A. Integrated Metagenomic and Metabolomic Analysis of In Vitro Murine Gut Microbial Cultures upon Bisphenol S Exposure. *Metabolites* **2024**, *14*, 713. https://doi.org/10.3390/metabo14120713.
6. Ma, X.; Cai, D.; Chen, Q.; Zhu, Z.; Zhang, S.; Wang, Z.; Hu, Z.; Shen, H.; Meng, Z. Hunting Metabolic Biomarkers for Exposure to Per- and Polyfluoroalkyl Substances: A Review. *Metabolites* **2024**, *14*, 392. https://doi.org/10.3390/metabo14070392.
7. Li, X.; Niu, H.; Huang, Z.; Zhang, M.; Xing, M.; Chen, Z.; Wu, L.; Xu, P. Deciphering the Role of the Gut Microbiota in Exposure to Emerging Contaminants and Diabetes: A Review. *Metabolites* **2024**, *14*, 108. https://doi.org/10.3390/metabo14020108.
8. Weng, Y.; Xu, T.; Wang, C.; Jin, Y. Oral Exposure to Epoxiconazole Disturbed the Gut Micro-Environment and Metabolic Profiling in Male Mice. *Metabolites* **2023**, *13*, 522. https://doi.org/10.3390/metabo13040522.
9. Mo, Z.; Wang, J.; Meng, X.; Li, A.; Li, Z.; Que, W.; Wang, T.; Tarnue, K.F.; Ma, X.; Liu, Y.; et al. The Dose-Response Effect of Fluoride Exposure on the Gut Microbiome and Its Functional Pathways in Rats. *Metabolites* **2023**, *13*, 1159. https://doi.org/10.3390/metabo13111159.
10. Feng, J.; Peng, J.; Hsiao, Y.-C.; Liu, C.-W.; Yang, Y.; Zhao, H.; Teitelbaum, T.; Wang, X.; Lu, K. Non/Low-Caloric Artificial Sweeteners and Gut Microbiome: From Perturbed Species to Mechanisms. *Metabolites* **2024**, *14*, 544. https://doi.org/10.3390/metabo14100544.
11. Chu, X.; Xing, H.; Chao, M.; Xie, P.; Jiang, L. Gut Microbiota Modulation in Osteoporosis: Probiotics, Prebiotics, and Natural Compounds. *Metabolites* **2025**, *15*, 301. https://doi.org/10.3390/metabo15050301.

Regarding the effects of microplastics, Contribution 1 found that polystyrene (PS) microplastic exposure (0.1 mg/day) perturbed the gut microbiota composition, diversity, and functional pathways in C57BL/6 mice. Metabolomic analysis revealed that cholesterol metabolism, primary and secondary bile acid biosynthesis, taurine and hypotaurine metabolism, as well as short-chain fatty acid metabolism, were perturbed upon microplastic exposure. Contribution 2 found that the impact of PS microplastics on the intestinal barrier function was different between mice fed a high-fat diet and a normal diet. Contribution 3 found that PS microplastics exacerbate high-fat diet-induced glucose metabolism disorder in male C57BL/6 mice. Moreover, Contribution 4 reviewed studies on the adverse effects of micro- and nanoplastics on invertebrates and vertebrates, as well as on the gut microbiota and its metabolites. Contribution 5 investigated the impact of BPS on gut microbiota using in vitro cultures and found that compared to controls, BPS did not overtly distort profiles of the metagenome and metabolome of the microbial cultures even at a supraphysiologic dose. Contribution 6 summarized recent metabolomics-based studies and elucidated the potential biomarkers of PFAS exposure. Contribution 7 discussed the link between emerging pollutants and glucose metabolism and the potential role played by the gut microbiota. These studies provide insights into the toxicity of emerging contaminants and the underlying mechanisms associated with microbial changes.

In addition to emerging contaminants, the effects of traditional contaminants on gut microbiota were also investigated in our Special Issue. EPX is a triazole fungicide, which is widely used to control pests in agriculture. Contribution 8 found that EPX exposure disrupted intestinal barrier function and altered the composition and abundance of gut microbiota and the glycolipid metabolism in male C57BL/6 mice. Contribution 9 found fluoride exposure induced a profound shift in the gut microbial composition, with dose-dependent responses observed in key genera. Microbial functional pathways were also disturbed by fluoride exposure, including D-lyxose ketol-isomerase and DNA polymerase III subunit gamma/tau.

Dietary factors are key factors that influence the gut microbiota. NAS are chemical additives that substitute sugars to avoid sugar-derived diseases. Contribution 10 implemented a comprehensive two-stage literature analysis and found that specific NAS exhibited discrepant impacts on the gut microbiota, and the overlapping microbial genera and species were identified. Impairment of glucose tolerance in the host was induced by some NAS, but the key metabolites and underlying mechanisms varied. Contribution 11 reviewed the recent findings on the role of prebiotics, probiotics, and natural bioactive substances in the modulation of gut microbiota to improve the health of the bone.

In conclusion, this Special Issue of *Metabolites* highlights the significant progress made in understanding the complex interactions between environmental exposures, host metabolism, and microbial communities. These studies are pivotal in unraveling the intricate metabolic processes that shape both human health and the dynamics of our microbial partners. However, further research is essential to elucidate the causal roles that gut microbiota may play in disease development under varying environmental conditions. The findings presented here underscore the need for continued investigation into these interdependencies, ultimately guiding the development of more targeted and effective strategies for disease prevention and treatment.

